# Understanding the Impact of Key Wine Components on the Use of a Non-Swelling Ion-Exchange Resin for Wine Protein Fining Treatment

**DOI:** 10.3390/molecules26133905

**Published:** 2021-06-26

**Authors:** Lin Sun, Ananya Srinivas, Ron C. Runnebaum

**Affiliations:** 1 Department of Chemical Engineering, University of California, Davis, CA 95616, USA; sunlinaz699@gmail.com (L.S.); anysrinivas@ucdavis.edu (A.S.); 2 Department of Viticulture & Enology, University of California, Davis, CA 95616, USA

**Keywords:** wine, protein, ion-exchange resin, fining

## Abstract

The impact of key classes of compounds found in wine on protein removal by the ion-exchange resin, Macro-Prep^®^ High S, was examined by adsorption isotherm experiments. A model wine system, which contained a prototypical protein Bovine Serum Albumin (BSA), was used. We systematically changed concentrations of individual chemical components to generate and compare adsorption isotherm plots and to quantify adsorption affinity or capacity parameters of Macro-Prep^®^ High S ion-exchange resin. The pH (hydronium ion concentration), ethanol concentration, and prototypical phenolics and polysaccharide compounds are known to impact interactions with proteins and thus could alter the adsorption affinity and capacity of Macro-Prep^®^ High S ion-exchange resin. At low equilibrium protein concentrations (< ~0.3 (g BSA)/L) and at high equilibrium protein concentrations in model wines at various pH, the adsorption behavior followed the Langmuir isotherm, most likely due to the resin acting as a monolayer adsorbent. The resulting range of BSA capacity was between 0.15–0.18 (g BSA)/(g Macro-Prep^®^ High S resin). With the addition of ethanol, catechin, caffeic acid, and polysaccharides, the protein adsorption behavior was observed to differ at higher equilibrium protein concentrations (> ~0.3 (g BSA)/L), likely as a result of Macro-Prep^®^ acting as an unrestricted multilayer adsorbent at these conditions. These data can be used to inform the design and scale-up of ion-exchange columns for removing proteins from wines.

## 1. Introduction

Bentonite has been used worldwide as the protein fining agent in winemaking and its use can be dated back more than 80 years ago [1]. In wine, the charge of proteins is determined by the relative relationship between pH and the isoelectric point (pI) of the protein. Proteins are positively charged when pH is below their pI. Bentonite provides a negatively charged surface to adsorb proteins by cation exchange. It is a mature technology to achieve protein stability of wine by adding sodium (or calcium) bentonite [2,3].

There are some drawbacks, however, in the use of bentonite. The separation of bentonite from wine is difficult and time-consuming. It can also cause significant loss of wine because about 3–10% of wine is absorbed by the bentonite slurry due to its significant swelling effect when hydrated in wine [4]. The use of bentonite can lead to sub-optimal working conditions because of the potential for dust (e.g., silica) generation and create additional downstream processing to remove the solids remaining dispersed in wine [5]. Due to its low density, bentonite takes 1–2 weeks of time to settle by gravity to the bottom of the tank. Thus, developing environmentally friendly alternatives to reduce both residues and wine loss has become vitally important so that wineries can minimize time and effort spent on removing bentonite residues in downstream processes and not contribute to creating more liquid and solid waste.

Fining agents, which can be regenerated and reused, can be developed to remove wine proteins more efficiently by reducing the time required and minimizing wine loss. Synthetic ion-exchange materials, such as resins developed for bio-tech once-through, packed-bed process operations [6], have shown reasonable affinity and capacity for wine proteins in model wine solutions at bench-scale [5,7,8]. The physical and chemical principles—those of ion-exchange—are at work in these synthetic materials and in bentonite. The use of an approach based on identical chemical and physical principles would facilitate more seamless integration, in contrast to using membranes, for instance, which can also remove additional large molecules such as polysaccharides and lead to other instabilities after bottling. While synthetic ion-exchange materials can be regenerated and reused in the pharmaceutical applications for which they were designed, data determining the stability of ion-exchange resins after use and regeneration in wine applications are lacking.

Macro-Prep^®^ High S by Bio-Rad is a non-swelling ion-exchange cellulose particle that has been proposed to be used as a bentonite alternative [8,9,10]. It is a mature bio-tech application for protein purification as proteins can freely bind to and release from the outer surface of the Macro-Prep^®^ particles especially by changing process conditions between adsorption and desorption. Macro-Prep^®^ High S is a strong cation exchanger that contains sulfonate functional groups; the working hypothesis is that proteins can be adsorbed onto its surface from wine.

The approach will be to determine adsorption isotherm curve profiles for model wine solutions, which consist of different chemical components that are used to mimic a real wine. Because the sulfonate functional groups of Macro-Prep^®^ create a fixed number of cation exchange sites, it is anticipated that the adsorption isotherm and the fraction of sites occupied will be consistent with the Langmuir isotherm equation. The Langmuir isotherm explains the equilibrium found in adsorption with a finite number of sites at the monolayer level [11]. The equilibrium between proteins in model wine solution and Macro-Prep^®^ is displayed by the Langmuir adsorption isotherm. The adsorption of protein from model wine solution by Macro-Prep^®^ particles has been shown to be consistent with the assumption of the Langmuir isotherm equation [8]. Changes in the adsorption isotherms will be interpreted by determining parameters for adsorption affinity and capacity and by comparing to values that characterize adsorption from a simple model wine solution in the absence of additional prototypical wine compounds (e.g., phenolics).

To characterize the adsorption affinity and capacity of Macro-Prep^®^ High S in the presence of prototypical components of wine, a model protein such as Bovine Serum Albumin (BSA) can be used to quantify these adsorption parameters from model wine solutions [10]. BSA, which has a molecular weight of around 66.5 kDa, is moderately non-reactive and has good solubility in water. It has been shown to have representative protein–phenolics interactions, albeit it under higher pH conditions (e.g., 5–7.5 pH) than present in wine (e.g., 3–4 pH), including with small phenolics such as caffeic acid and catechin [12,13,14]. Interactions between proteins and other wine compounds, such as phenolics, have also been reported under conditions relevant to wine consumption; these often focus on proteins found in saliva or used for wine treatment [15,16]. BSA is chosen as a prototypical protein source because its isoelectric point (pI) in water is 4.3–4.8 [17,18,19]; therefore, BSA is positively charged at the pH of wines, ranging from 3.4 to 3.8. The size and pI of BSA is, therefore, characteristic and representative of fractions that are present naturally in wine. BSA has been used as a prototypical protein in wine-like systems [17,18].

A potential benefit in using solid ion-exchange materials is that their use can be scaled in continuous processing to achieve efficiencies in time, wine loss, and water usage (for cleaning). The scale-up and commercialization of synthetic ion-exchange materials have been accomplished for non-wine applications. More recently, metal oxides such as zirconium oxide have been evaluated and have also shown promise. However, scaling-up, even for other protein-based applications, to a continuous process has not been successful due to lack of adsorption in pellet-form and difficulties in high-temperature regeneration [20]. Regeneration protocols for winemaking applications need to be established, however, because wine contains ions and compounds not found in these other applications; work to determine the ability of these materials to be regenerated and reused needs to also be completed. To achieve the full benefits of solid materials, their translations to flow-through processes should be more thoroughly investigated.

In this work, we hypothesize that key classes of compounds found in wine will not have a significantly negative impact on adsorption, particularly under conditions of use for winemaking application. We therefore examine the impact of pH, ethanol concentration, prototypical phenolics and polysaccharide compounds to quantify their impact on adsorption affinity and capacity of Macro-Prep^®^ High S ion-exchange resin. By systematically changing individual chemical components (variables), we generate and compare isotherm plots and quantify adsorption affinity or capacity parameters of Marco-Prep^®^ High S ion-exchange resin.

## 2. Materials and Methods

### 2.1. Preparation of Model Wines

Protein-free model wine was prepared as follows in a 1 L flask: add 2 g of Potassium L-tartrate monobasic (KHT) (Sigma-Aldrich, USA) in 600 mL ultrapure deionized water (>18.0 MΩ resistivity) (Millipore, USA); mix the solution for 20 min until the KHT is dissolved; add 127 mL of 95% ethanol into the solution; add ultrapure deionized water into the volumetric flask until the water reaches the 1 L mark. Mix the solution for 20 min.

Model wine containing protein (1 g/L BSA) was prepared as follows in a 1 L Erlenmeyer flask: add 2 g of Potassium L-tartrate monobasic (KHT) (Sigma-Aldrich, USA) in 600 mL ultrapure deionized water; mix the solution for 20 min until KHT is dissolved; add 127 mL of 95% ethanol into the solution. Separately, in a 200 mL beaker, dissolve completely 1 g of OmniPur ^®^ Bovine Serum Albumin (BSA) (Sigma-Aldrich, USA) in 100 mL ultrapure deionized water; transfer the BSA protein solution into the 1 L volumetric flask; add ultrapure deionized water (>18.0 MΩ resistivity) (Millipore, USA) into the volumetric flask until the water reaches the 1 L mark. Mix the solution for 20 min.

Adjustments to pH were made with small amounts of 1 M HCl (Millipore, USA) or 1 M NaOH (Sigma-Aldrich, USA). Ethanol concentrations were adjusted by changing the volume of 95% ethanol added into the solutions. Prototypical compounds representing important classes of compounds found in wines were added at the concentrations specified in Table 1, including caffeic acid (Sigma-Aldrich, USA), catechin (Sigma-Aldrich, USA) and arabinogalactan (Spectrum Chemical, USA).

### 2.2. Experimental Design

The following variables were varied independently in the model wine solutions containing protein Bovine Serum Albumin (BSA): pH, ethanol concentration, caffeic acid (prototypical phenolic compound), catechin (prototypical phenolic compound), and arabinogalactan (prototypical polysaccharide), shown in Table 1. For pH, ethanol concentration, and arabinogalactan variations, the concentrations of the protein were measured with a UV-Vis spectrometer at 280 nm as well as by using the Bradford protein assay along with a UV-Vis spectrometer at 595 nm. However, due to the phenolic ring structure of catechin and caffeic acid, which also absorb at 280 nm, the concentrations of proteins in experiments with these two compounds could only be quantified by using the Bradford protein assay. Three different values of pH and ethanol were used, while two values of caffeic acid, catechin, and arabinogalactan were investigated. The quantitative values for these independent variables are found in Table 1.

### 2.3. Adsorption Isotherms

Macro-Prep^®^ High S resin (Bio-Rad, USA) was dried in an oven at 35 °C for 24 h to enable more accurate quantification of the mass use to prepare the model wine solutions. Before transferring the resin into wine solution, the dried Macro-Prep^®^ High S was weighed and subsequently rehydrated with 10 mL ultrapure deionized water for 10 min so that the rehydration condition of the resin is consistent. The resin was then placed into a 250 mL Erlenmeyer flask along with 150 mL of the previously prepared model wine solution (e.g., with a composition of 0.5 g/L OmniPur ^®^ Bovine Serum Albumin (BSA) (Sigma-Aldrich, USA), 2 g/L Potassium L-tartrate monobasic (KHT) (Sigma-Aldrich, USA), 12 *v*/*v* % ethanol). Erlenmeyer flasks were placed onto a shaker table with the rotation speed set to 220 rpm for 4 h at room temperature. To collect samples for characterization, syringes and syringe filters (HPLV, 0.45 μm, VWR International, USA) were used to remove solid particles and clarify the model wine solution before subsequent characterization by UV-Vis and the Bradford assay. Samples from each Erlenmeyer flask were collected separately into a 15 mL centrifuge tube. Concentrations of BSA were determined using a UV-Vis spectrometer (ThermoScientific Genesys 10s UV-VIS, USA).

To prepare samples for characterization by the Bradford assay, a sample volume of 4.5 mL from each centrifuge tube was mixed with 0.9 mL Coomassie PlusTM Protein Assay Reagent (ThermoScientific, USA) in another 15 mL centrifuge tube. After 10 min at room temperature, samples were characterized by using the UV-Vis spectrometer at 595 nm.

## 3. Results and Discussion

### 3.1. Impact of pH on Protein Adsorption by Macro-Prep^®^

**Figure 1:**
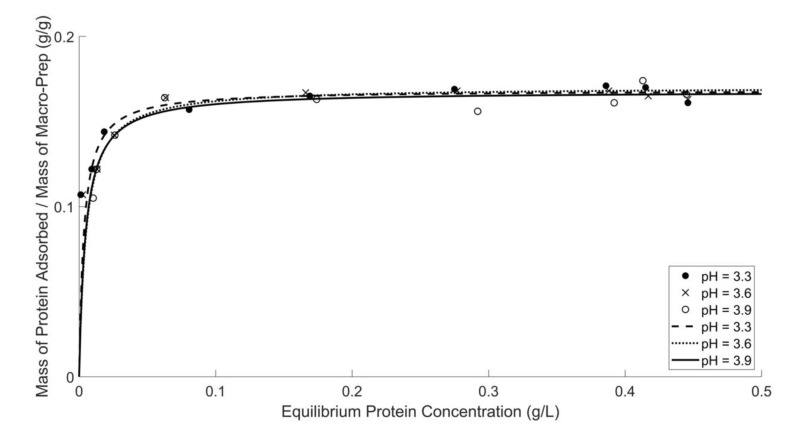
Protein adsorption by Macro-Prep^®^ in model wine solutions with different pH values. Protein concentration was measured by using the Bradford protein assay and a UV-Vis spectrometer at 595 nm.

The affinity and capacity of ion-exchange materials can be characterized by the mass of adsorbate, in this instance, bovine serum albumin (BSA) that is adsorbed from model wine solution per mass of adsorbent, in this instance, Macro-Prep^®^ High S resin. Equilibrium protein concentration data, defined as the protein concentration remaining in solution when the adsorption process reaches equilibrium conditions, were generated from experiments contacting protein-containing solutions with various amounts of Macro-Prep^®^. In Figure 1, it is shown that the protein adsorption isotherm of Macro-Prep^®^ High S is consistent with the model of Langmuir adsorption isotherm, which assumes that all ion-exchange sites are equal in size and shape on the surface of Macro-Prep^®^ resin. The complementary data are generated by probing the protein molecule directly by UV-Vis spectroscopy at 280 nm (Figure S1) and indirectly by reaction of the protein in the Bradford assay (Figure 1). These results also imply that adsorption is a monolayer under these experimental conditions. Comparing results collected from wine solutions of different pH, it can be found that the pH of wine solution did not significantly affect the protein adsorption ability of Macro-Prep^®^ High S resin.

**Figure 2:**
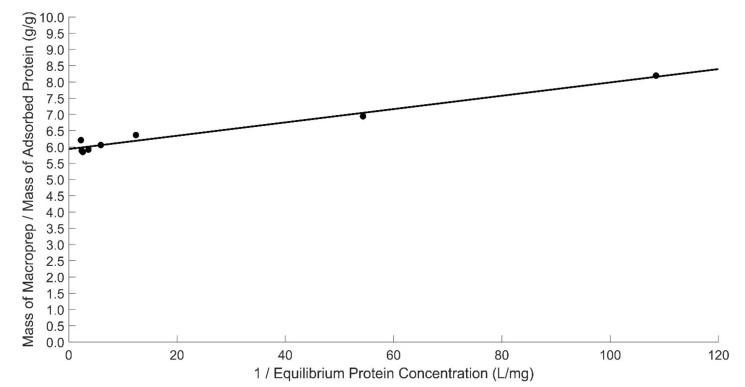
Lineweaver–Burk plot of Macro-Prep^®^ adsorption capacity in model wine solutions with pH of 3.3. Data collected by using the Bradford protein assay and a UV-Vis spectrometer at 595 nm.

A Lineweaver–Burk plot, shown in Figure 2 (and in Supporting Information Figures S2–S6, S8–S17, S19–S22), is used to determine the Langmuir constant and capacity for Macro-Prep^®^ in model wine. Table 2 reports the effect of pH on protein adsorption by displaying the Langmuir constant and Langmuir capacity when using Macro-Prep^®^ to remove BSA from model wine solutions with various pH values. Protein concentration was quantified by using both UV-Vis spectrometry at 280 nm and Bradford protein assay. Over the three pH values (3.3–3.9), the Langmuir capacity showed little change in both the UV-Vis and Bradford protein assays. Over the three pH values (3.3–3.9), the Langmuir constant also showed little change in affinity. The effect seems to be due to minimal competition between hydrogen ions and the protein in the lower pH solution.

### 3.2. Impact of Ethanol on Protein Adsorption by Macro-Prep^®^

**Figure 3:**
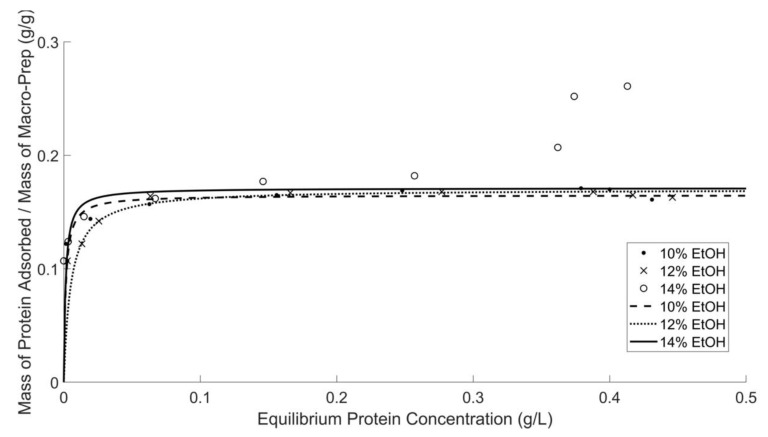
Macro-Prep^®^’s protein adsorption ability in model wine solutions with different ethanol concentrations by using the Bradford protein assay and UV-Vis spectrometer at 595 nm. Data for 14% *v/v* ethanol fitted only up to 0.3 (g BSA)/L.

Protein adsorption by Macro-Prep^®^, shown by the isotherm in Figure 3, is consistent with a Langmuir adsorption model for ethanol concentration of 10 and 12 *v*/*v* %; at high equilibrium concentrations, the adsorption from model wine solutions containing 14 *v*/*v* % ethanol deviates from the saturation of ion-exchange sites. A complementary adsorption isotherm by only using UV-Vis at 280 nm provided in Figure S7. A Langmuir isotherm assumes that all vacant ion-exchange sites are of equal size and shape on the surface and that adsorption is a monolayer; these assumptions may not be valid for Macro-Prep^®^ under experimental conditions of high equilibrium protein concentration in a 14 *v*/*v* % solution. Blade and Boulton assessed the characterization of bentonite at ethanol concentrations up to 13 *v*/*v* % and proposed that solvents such as ethanol can displace water molecules and lead to more extensive swelling [18]. The result can be increased access and adsorption of proteins. Their work did not graphically report the adsorption isotherms; it is unclear therefore if their analysis included experiments at higher equilibrium concentrations where deviations are observed with Macro-Prep^®^. Data from both protein quantification methods (e.g., Bradford Assay and direct characterization by UV-Vis at 280 nm) in this work suggest that the ethanol concentration of wine solution does not significantly affect the protein adsorption ability of Macro-Prep^®^ High S resin, except at the highest equilibrium concentrations in the 14 *v*/*v* % ethanol model wine in which the capacity is enhanced.

Adsorption capacity and affinity are quantified, shown in Table 3, by determining these values from Lineweaver–Burk plots, shown in Supporting Information Figures S8–S13. Data collected by a UV-Vis spectrometer at 280 nm show that a 0.16–0.18 (g BSA)/(g Macro-Prep^®^ High S resin) is adsorbed from the model wine which contains 10–12% *v/v* ethanol. Results from the Bradford assay also show that the maximum protein capacity of Macro-Prep^®^ High S resin 0.16–0.17 (g BSA)/(g Macro-Prep^®^ High S resin) from wine which contains 10–12 *v*/*v* % ethanol. At 14 *v*/*v* % ethanol, the protein adsorption capacity at high equilibrium concentration deviates from approaching a maximum value; at more moderate equilibrium concentrations, up to 0.15 (g BSA)/L, the adsorption capacity approaches 0.16 and 0.17 (g BSA)/(g Macro-Prep^®^ High S resin) for UV-Vis at 280 nm and by the Bradford Assay, respectively.

Compared to bentonite, the impact of change in ethanol concentrations appears to have less of an impact on Langmuir capacity and affinity of Macro-Prep^®^ High S resin. As ethanol concentration increases from 10 to 13 *v/v* % ethanol, the Langmuir capacity of bentonite increases slightly from 0.689 to 0.780 [9,18]. Comparatively, the isotherm graphs for Macro-Prep^®^ qualitatively also display a slight increase in protein adsorption capacity as ethanol concentration increases for low equilibrium protein conditions.

### 3.3. Impact of Prototypical Phenolic Compounds, Caffeic Acid and Catechin, on Protein Adsorption by Macro-Prep^®^

**Figure 4:**
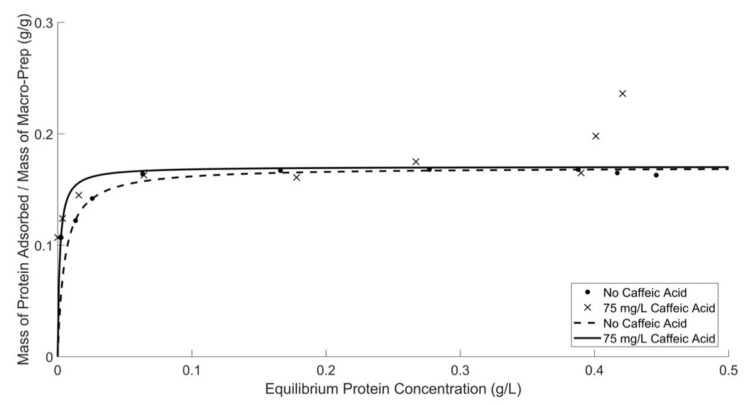
Macro-Prep^®^’s protein adsorption ability in model wine solutions with and without the addition of caffeic acid by using the Bradford protein assay and UV-Vis spectrometer at 595 nm. Caffeic acid fit excludes the two data points at high equilibrium protein concentrations, >0.4 (g BSA)/L. When fitting only these data, the adsorption capacities are not significantly different from each other.

The protein adsorption isotherm of Macro-Prep^®^ in wine solution with and without caffeic acid, shown in Figure 4, are consistent with the Langmuir adsorption isotherm model, apart from high equilibrium concentrations (>0.4 (g BSA)/L) for the solution containing caffeic acid. At higher equilibrium concentrations, the presence of prototypical phenolic compounds, caffeic acid, enables Macro-Prep^®^ to act as a multilayer protein adsorbent, contrasted by the observed results at lower equilibrium conditions that are consistent with monolayer adsorption. Caffeic acid is likely enabling longer-range interactions with BSA [13,14], when BSA is present at the higher equilibrium concentrations used in this work. Only the Bradford protein assay was applied in this experiment to quantify the concentration of protein dissolved in the wine because caffeic acid absorbs in the UV spectrum at the wavelength of 280 nm.

Adsorption capacity and affinity are quantified, shown in Table 4, by determining these values from Lineweaver–Burk plots, shown in Supporting Information Figures S14 and S15. Data collected by the Bradford assay show that a 0.17 (g BSA)/(g Macro-Prep^®^ High S resin) is adsorbed at low equilibrium protein concentrations (<0.4 (g BSA)/L), which is not different from the capacity of Macro-Prep^®^ High S in the absence of caffeic acid.

**Figure 5:**
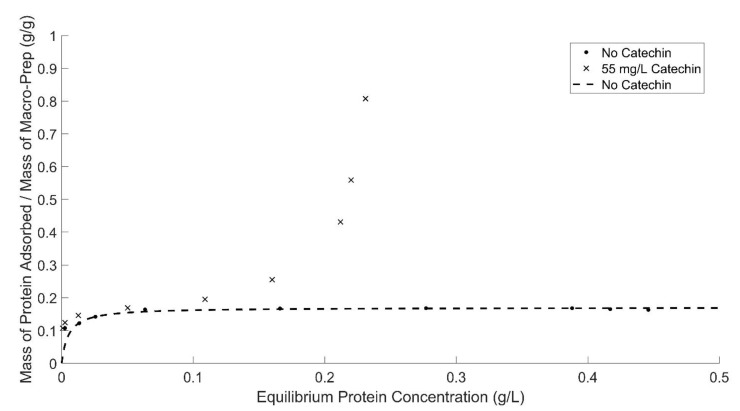
Macro-Prep^®^’s protein adsorption ability in model wine solutions with and without the addition of catechin by using the Bradford protein assay and UV-Vis spectrometer at 595 nm. The fit deviates from assumptions for Langmuir isotherm at high equilibrium concentrations of BSA. When fitting only the four lowest equilibrium concentrations of BSA, the adsorption capacity of Macro-Prep^®^ High S in the presence of catechin is about 10% lower than in the absence of catechin. A Langmuir isotherm fit with the addition of catechin is not shown.

The adsorption capacity of Macro-Prep^®^, shown in Figure 5, is changed with the addition of catechin into the model wine solution, especially at high equilibrium protein concentrations (when less Macro-Prep^®^ resin is added into the solution). To reduce the influence of catechin on quantification, only the Bradford protein assay was applied in this experiment to quantify the concentration of protein dissolved in the wine. It can be found that the addition of catechin can affect Macro-Prep^®^’s protein adsorption ability because the nature of the adsorption isotherm is changed to model the kind of isotherm observed for unrestricted mono-multilayer solid adsorbents. Typically, at low equilibrium concentrations, this kind of adsorbate-adsorbent interaction acts as monolayer material and at higher equilibrium concentrations the solid has multilayer adsorption ability. Catechin is likely creating interactions with BSA [13,14] at high equilibrium concentrations that enable longer-range interactions with more than a monolayer of protein adsorbed to the surface. The Macro-Prep^®^ High S resin is a macroporous material and, in this instance, behaves as a mono-multilayer solid adsorbent when 55 mg of catechin is added into 1 L of wine solution.

Adsorption capacity and affinity by Macro-Prep^®^ in the presence of catechin are quantified, shown in Table 5, by determining these values from Lineweaver–Burk plots, shown in Supporting Information Figures S16 and S17. Data collected by the Bradford assay show that a 0.15 (g BSA)/(g Macro-Prep^®^ High S resin) is adsorbed at low equilibrium protein concentrations (<0.4 (g BSA)/L). This decrease in capacity of about 10%, which is dependent upon the number of data points used in the model, will be an important consideration when equilibrium protein concentrations are required for treatment with Macro-Prep^®^ High S in the presence of compounds such as catechin.

### 3.4. Impact of a Prototypical Polysaccharide on Protein Adsorption by Macro-Prep^®^

**Figure 6:**
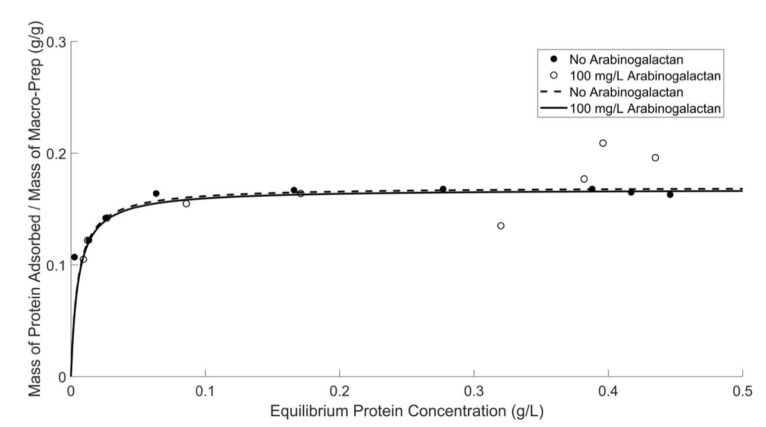
Macro-Prep^®^’s protein adsorption ability in model wine solutions with and without the addition of arabinogalactan (AG) by using the Bradford protein assay and a UV-Vis spectrometer at 595 nm. Data for 100 mg/L of arabinogalactan are fitted only up to 0.17 (g BSA)/L.

The adsorption capacity of Macro-Prep^®^, shown in Figure 6, is not impacted by the addition of arabinogalactan into model wine solution, especially at lower equilibrium protein concentrations. It is shown that the addition of arabinogalactan, used as a model polysaccharide, can have an influence on Macro-Prep^®^’s protein adsorption ability at high equilibrium protein concentrations. These results also imply that adsorption may deviate slightly from monolayer at higher equilibrium protein experimental conditions. Data for 100 mg/L of arabinogalactan are fitted, therefore, only up to 0.17 (g BSA)/L.

The effect of arabinogalactan on protein adsorption is shown in Table 6, including the Langmuir constant and Langmuir capacity for solutions with and without arabinogalactan by using both a UV-Vis spectrometer at 280 nm and the Bradford protein assay. These values were determined from Lineweaver–Burk plots, shown in Supporting Information Figures S19–S22. When 100 mg of arabinogalactan is added into model wine solution with 0.5 g/L of BSA, every 0.16–0.17 g of BSA can be adsorbed by 1 g of Macro-Prep^®^ High S resin. In this case, the maximum protein adsorption capacity of Macro-Prep^®^ High S resin is estimated to change negligibly.

## 4. Conclusions

In conclusion, the Macro-Prep^®^ High S resin has a stable performance (i.e., adsorption capacity and affinity) in different wine-like conditions. It is consistent with its potential use as a good replacement for bentonite as a protein fining agent. The protein adsorption capacity of Macro-Prep^®^ High S resin at low protein equilibrium concentrations is negligibly affected by changing pH, changing ethanol concentration, or adding chemicals that represent compound classes commonly found in wine, such as caffeic acid and the model polysaccharide compound arabinogalactan. Typically, the protein adsorption behavior of Macro-Prep^®^ High S follows the model of a Langmuir adsorption isotherm under these conditions.

At high equilibrium protein concentrations (>0.3 (g BSA)/L), the protein adsorption behavior by Macro-Prep^®^ changes with the addition of caffeic acid and polysaccharides and at higher ethanol concentrations. Macro-Prep^®^ High S behaves differently under these conditions, likely due to Macro-Prep^®^ acting as an unrestricted multilayer adsorbent. When the Macro-Prep^®^ resin is in contact with a wine solution with low equilibrium protein concentration, Macro-Prep^®^ will generally act as a monolayer adsorbent; however, when catechin is present, the adsorption behavior deviates from a Langmuir isotherm at low equilibrium protein concentrations of less than 0.2 (g BSA)/L. If the protein concentration in the wine solution is high under equilibrated conditions with the Macro-Prep^®^, the adsorption on the surface of the Macro-Prep^®^ solid can reach saturation and the monolayer coverage reaches a maximum. This point is a critical point where Macro-Prep^®^ can be taken as a mono-multilayer adsorbent. Above this critical point, Macro-Prep^®^ solids, in the presence of wine compounds including caffeic acid and the model polysaccharide arabinogalactan, act as multilayer protein adsorbents. In this situation, both inter-molecule and intra-molecule adsorption can be observed simultaneously.

Differences in adsorption capacity between Macro-Prep^®^ High S and bentonite, as well as any deviations from Langmuir isotherm behavior, can be used to inform design processes that use packed bed adsorption column for removing proteins from wines.

## Figures and Tables

**Table 1:** Experimental conditions varied in the model wine solutions containing protein Bovine Serum Albumin (BSA) ^1^.

	Min	Mid	Max
pH	3.3	3.6	3.9
Ethanol (*v*/*v* %)	10	12	14
Caffeic acid (mg/L)	0		75
Catechin (mg/L)	0		55
Arabinogalactan (mg/L)	0		100

^1^ Protein-free model wine and protein model wine were combined to create model wines with 0.5 g/L BSA protein for each of these experimental conditions.

**Table 2:** Effect of pH on protein adsorption and removal from model wine solutions by the ion-exchange resin Macro-Prep^®^.

	Model Wine pH ^1^			Model Wine pH ^2^		
	3.3	3.6	3.9	3.3	3.6	3.9
Langmuir constant, K_L_ (g/L)	0.0041	0.0057	0.022	0.0035	0.0051	0.0047
Langmuir capacity, (q_m_) (g/g)	0.17	0.16	0.16	0.17	0.17	0.17
Langmuir correlation coefficient	0.97	0.98	0.89	0.98	0.99	0.96

^1^ Protein concentration determined using a UV-Vis spectrometer at 280 nm. ^2^ Protein concentration determined using the Bradford protein assay by and a UV-Vis spectrometer at 595 nm.

**Table 3:** Effect of ethanol concentration on protein adsorption and removal from model wine solutions by the ion-exchange resin Macro-Prep^®^.

	Ethanol Concentration (*v*/*v* %) ^1^			Ethanol Concentration (*v*/*v* %) ^2^		
	10%	12%	14%	10%	12%	14%
Langmuir constant, K_L_ (g/L)	0.033	0.0060	0.0017	0.0014	0.0051	0.0013
Langmuir capacity, (q_m_) (g/g)	0.18	0.16	0.16	0.16	0.17	0.17
Langmuir correlation coefficient	0.97	0.99	0.97	0.98	0.99	0.94

^1^ Protein concentration determined using a UV-Vis spectrometer at 280 nm. ^2^ Protein concentration determined using the Bradford protein assay by and a UV-Vis spectrometer at 595 nm.

**Table 4:** Effect of caffeic acid on protein adsorption from a model wine solution. Protein concentration was monitored by using the Bradford protein assay and UV-Vis spectrometry at 595 nm.

Caffeic Acid (mg/L)	0	75
Langmuir constant, K_L_ (g/L)	0.0051	0.0015
Langmuir capacity, (q_m_) (g/g)	0.17	0.17
Langmuir correlation coefficient	0.99	0.89

**Table 5:** Effect of catechin on protein adsorption. Protein concentration was monitored by using the Bradford protein assay and UV-Vis spectrometry at 595 nm. The Langmuir isotherm parameters in the presence of catechin are estimated for low protein equilibrium concentrations.

Catechin (mg/L)	0	55
Langmuir constant K_L_ (g/L)	0.0051	0.00038
Langmuir capacity (q_m_) (g/g)	0.17	0.15
Langmuir correlation coefficient	0.99	0.94

**Table 6:** Effect of prototypical polysaccharide on protein adsorption. The Langmuir isotherm parameters in the presence of arabinogalactan are estimated for low protein equilibrium concentrations.

	Arabinogalactan (mg/L) ^1^		Arabinogalactan (mg/L) ^2^	
	0	100	0	100
Langmuir constant K_L_ (g/L)	0.0060	0.0038	0.0051	0.0052
Langmuir capacity (q_m_) (g/g)	0.16	0.16	0.17	0.17
Langmuir correlation coefficient	0.99	0.93	0.99	0.99

^1^ Protein concentration determined using a UV-Vis spectrometer at 280 nm. ^2^ Protein concentration determined using the Bradford protein assay by and a UV-Vis spectrometer at 595 nm.

## Data Availability

The data presented in this study are available within the article or supplementary material.

## Supplementary Materials

The following are available online, Figure S1: Protein adsorption by Macro-Prep in model wine solutions with different pH values. Protein concentrations were measured by using a UV-Vis spectrometer at 280 nm. Figure S2: Lineweaver-Burk plot of Macro-Prep adsorption capacity in model wine solutions with pH of 3.3. Data collected by using a UV-Vis spectrometer at 280 nm. Figure S3: Lineweaver-Burk plot of Macro-Prep adsorption capacity in model wine solutions with pH of 3.6. Data collected by using a UV-Vis spectrometer at 280 nm. Figure S4: Lineweaver-Burk plot of Macro-Prep adsorption capacity in model wine solutions with pH of 3.6. Data collected by using the Bradford protein assay and a UV-Vis spectrometer at 595 nm. Figure S5: Lineweaver-Burk plot of Macro-Prep adsorption capacity in model wine solutions with pH of 3.9. Data collected by using a UV-Vis spectrometer at 280 nm. Figure S6: Lineweaver-Burk plot of Macro-Prep adsorption capacity in model wine solutions with pH of 3.9. Data collected by using the Bradford protein assay and a UV-Vis spectrometer at 595 nm. Figure S7: Macro-Prep’s protein adsorption ability in model wine solutions with different ethanol concentrations characterized by UV-Vis spectrometer at 280 nm. Data for 14% *v/v* ethanol fitted only up to 0.17 (g BSA)/L. Figure S8: Lineweaver-Burk plot of Macro-Prep adsorption capacity in model wine solutions with an ethanol concentration of 10%. Data collected by using a UV-Vis spectrometer at 280 nm. Figure S9: Lineweaver-Burk plot of Macro-Prep adsorption capacity in model wine solutions with an ethanol concentration of 10%. Data collected by using the Bradford protein assay and a UV-Vis spectrometer at 595 nm. Figure S10: Lineweaver-Burk plot of Macro-Prep adsorption capacity in model wine solutions with an ethanol concentration of 12%. Data collected by using a UV-Vis spectrometer at 280 nm. Figure S11: Lineweaver-Burk plot of Macro-Prep adsorption capacity in model wine solutions with an ethanol concentration of 12%. Data collected by using the Bradford protein assay and a UV-Vis spectrometer at 595 nm. Figure S12: Lineweaver-Burk plot of Macro-Prep adsorption capacity in model wine solutions with an ethanol concentration of 14%. Data collected by using a UV-Vis spectrometer at 280 nm. Figure S13: Lineweaver-Burk plot of Macro-Prep adsorption capacity in model wine solutions with an ethanol concentration of 14%. Data collected by using the Bradford protein assay and a UV-Vis spectrometer at 595 nm. Figure S14: Lineweaver-Burk plot of Macro-Prep adsorption capacity in model wine solutions with no addition of caffeic acid. Data collected by using the Bradford protein assay and a UV-Vis spectrometer at 595 nm. Figure S15: Lineweaver-Burk plot of Macro-Prep adsorption capacity in model wine solutions with addition of 75 mg/L caffeic acid. Data collected by using the Bradford protein assay and a UV-Vis spectrometer at 595 nm. Figure S16: Lineweaver-Burk plot of Macro-Prep adsorption capacity in model wine solutions with no addition of catechin. Data collected by using the Bradford protein assay and a UV-Vis spectrometer at 595 nm. Figure S17: Lineweaver-Burk plot of Macro-Prep adsorption capacity in model wine solutions with the addition of 55 mg/L catechin. Data collected by using the Bradford protein assay and a UV-Vis spectrometer at 595 nm. Figure S18: Macro-Prep’s protein adsorption ability in model wine solutions with & without addition of arabinogalactan (AG) by using a UV-Vis spectrometer at 280 nm. The presence of the prototypical polysaccharide does not have a negative impact on the adsorption affinity or capacity of the ion-exchange resin for the model protein, BSA. Data for 100 mg/L of arabinogalactan is fitted only up to 0.3 (g BSA)/L., Figure S19: Lineweaver-Burk plot of Macro-Prep adsorption capacity in model wine solutions with no addition of arabinogalactan (AG). Data collected by using a UV-Vis spectrometer at 280 nm. Figure S20: Lineweaver-Burk plot of Macro-Prep adsorption capacity in model wine solutions with no addition of arabinogalactan (AG). Data collected by using the Bradford protein assay and a UV-Vis spectrometer at 595 nm. Figure S21: Lineweaver-Burk plot of Macro-Prep adsorption capacity in model wine solutions with the addition of 100 mg/L arabinogalactan (AG). Data collected by using a UV-Vis spectrometer at 280 nm. Figure S22: Lineweaver-Burk plot of Macro-Prep adsorption capacity in model wine solutions with the addition of 100 mg/L arabinogalactan (AG). Data collected by using the Bradford protein assay and a UV-Vis spectrometer at 595 nm.

## Sample Availability

Samples of the materials are not available from the authors.
