# Supplementary material for: Understanding the Impact of Key Wine Components on the Use of a Non-Swelling Ion-Exchange Resin for Wine Protein Fining Treatment
Source: Molecules. 2021 Jun 26;26(13):3905. doi: 10.3390/molecules26133905 (PMC8272113; doi:10.3390/molecules26133905)
Supplement: Supplementary file 1 [file molecules-26-03905-s001.zip › molecules-1259300-supplementary.pdf]

molecules

MDPI

Supplementary Materials

Understanding the Impact of Key Wine Components on the Use of a Non-Swelling Ion-Exchange Resin for Wine Protein Fining Treatment

Lin Sun [1], Ananya Srinivas [1] and Ron C. Runnebaum [1,2,*]

[1] Department of Chemical Engineering, University of California, Davis, CA 95616, USA; sunlinaz699@gmail.com (L.S.); anysrinivas@ucdavis.edu (A.S.)
[2] Department of Viticulture & Enology, University of California, Davis, CA 95616, USA
* Correspondence: rcrunnebaum@ucdavis.edu; Tel: +1-530-752-9078

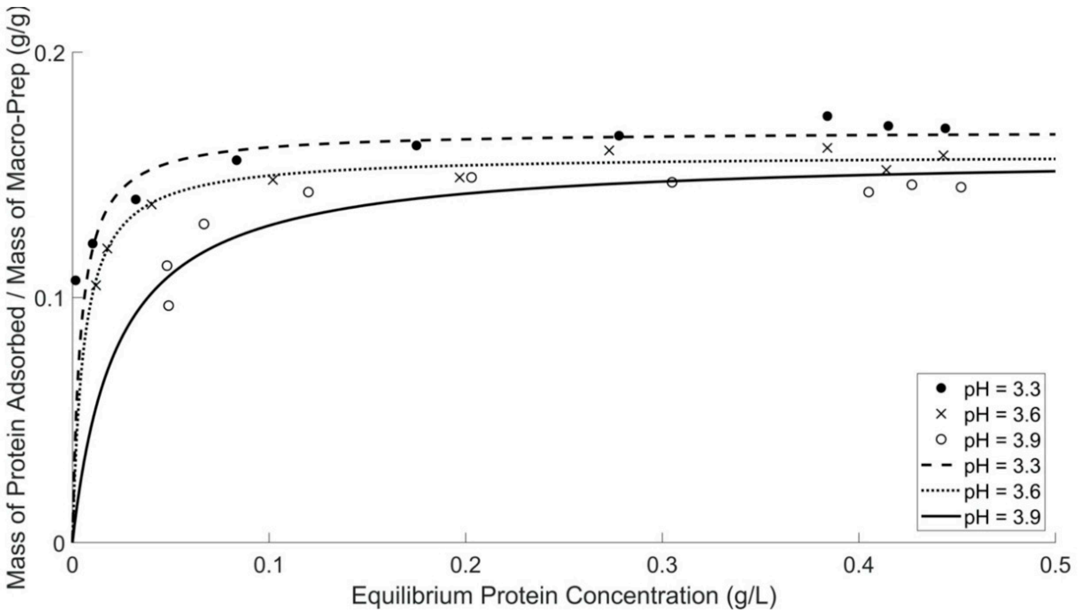

Figure S1. Protein adsorption by Macro-Prep in model wine solutions with different pH values. Protein concentrations were measured by using a UV-Vis spectrometer at 280 nm.

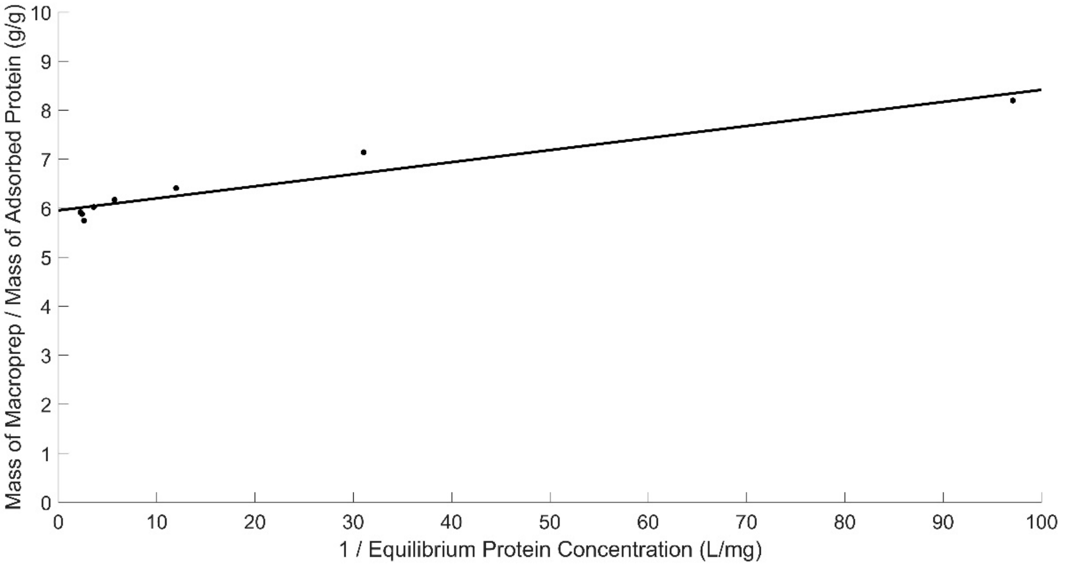

Figure S2. Lineweaver-Burk plot of Macro-Prep adsorption capacity in model wine solutions with pH of 3.3. Data collected by using a UV-Vis spectrometer at 280 nm.

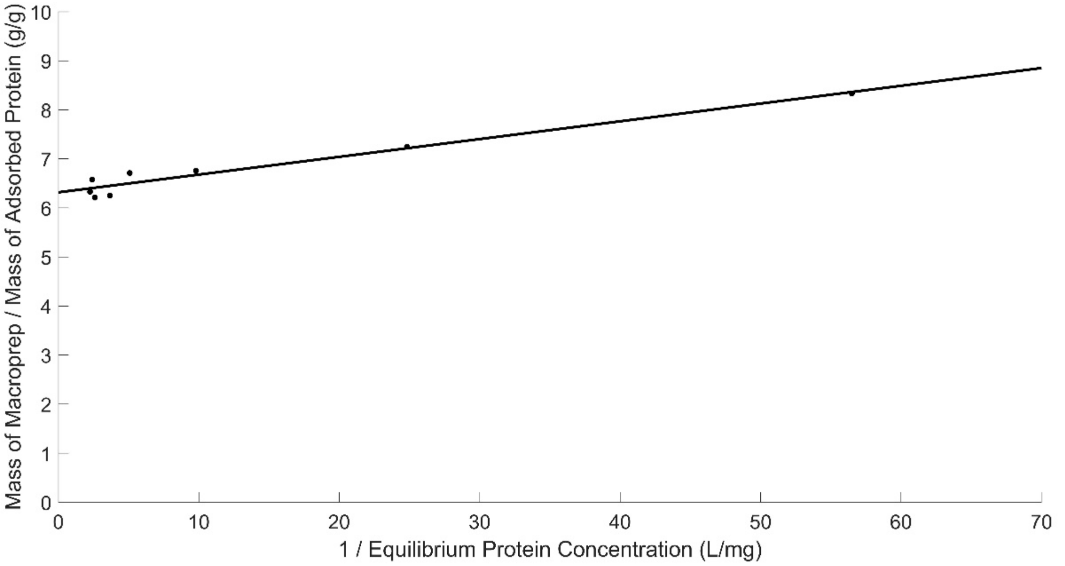

Figure S3. Lineweaver-Burk plot of Macro-Prep adsorption capacity in model wine solutions with pH of 3.6. Data collected by using a UV-Vis spectrometer at 280 nm.

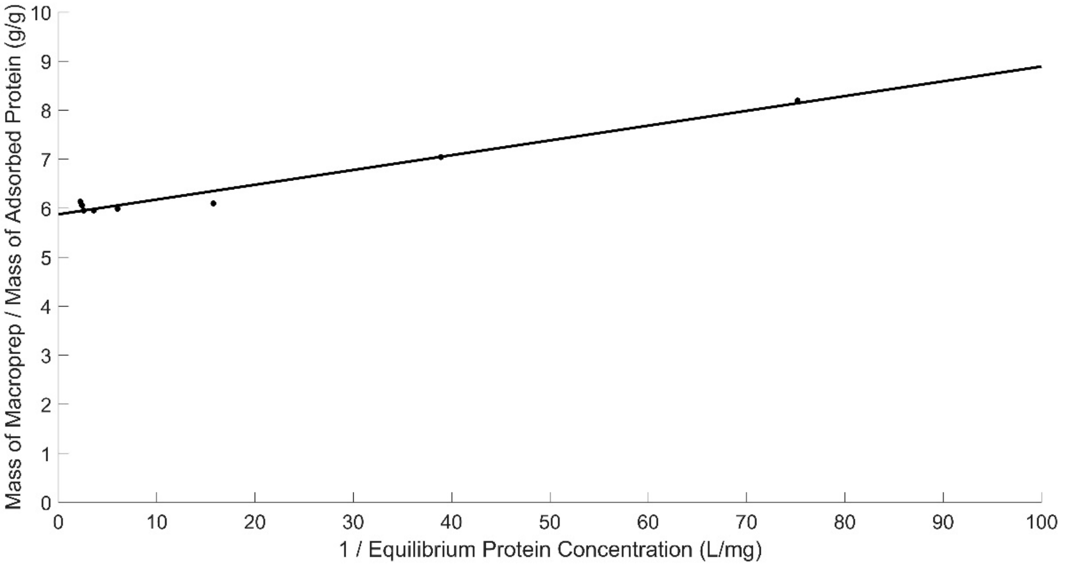

Figure S4. Lineweaver-Burk plot of Macro-Prep adsorption capacity in model wine solutions with pH of 3.6. Data collected by using the Bradford protein assay and a UV-Vis spectrometer at 595 nm.

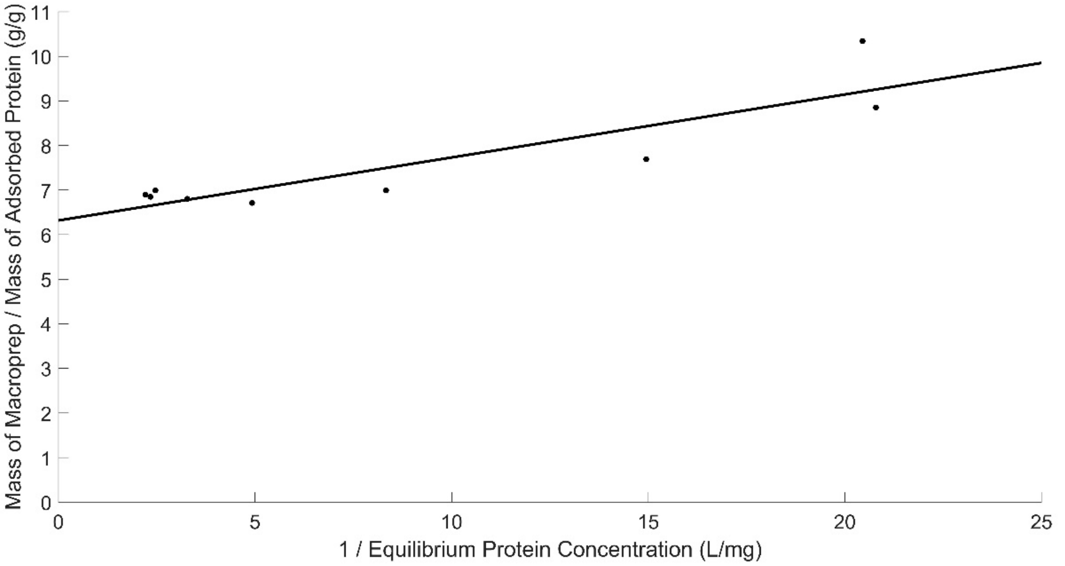

Figure S5. Lineweaver-Burk plot of Macro-Prep adsorption capacity in model wine solutions with pH of 3.9. Data collected by using a UV-Vis spectrometer at 280 nm.

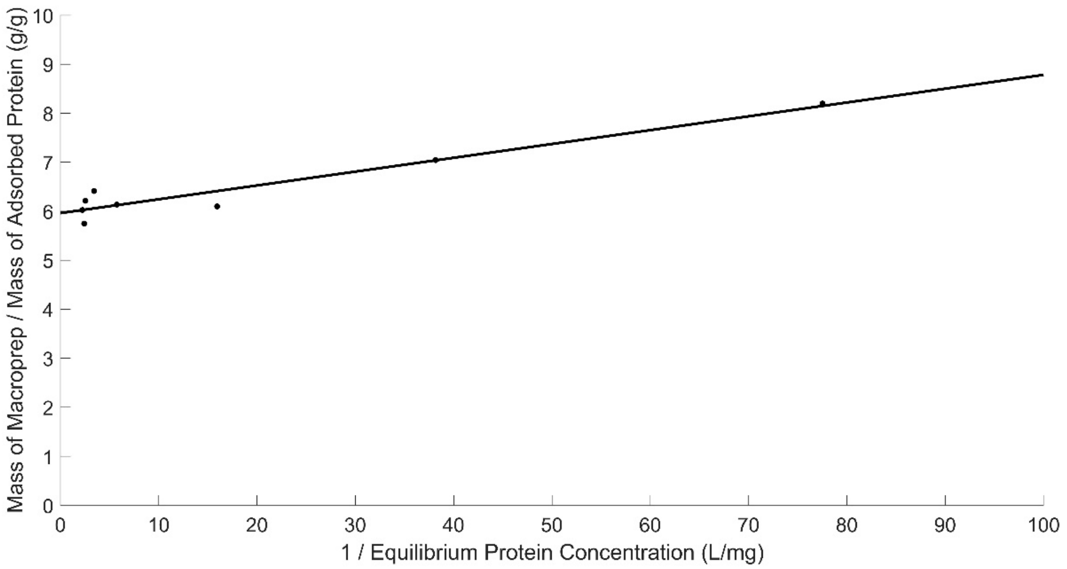

Figure S6. Lineweaver-Burk plot of Macro-Prep adsorption capacity in model wine solutions with pH of 3.9. Data collected by using the Bradford protein assay and a UV-Vis spectrometer at 595 nm.

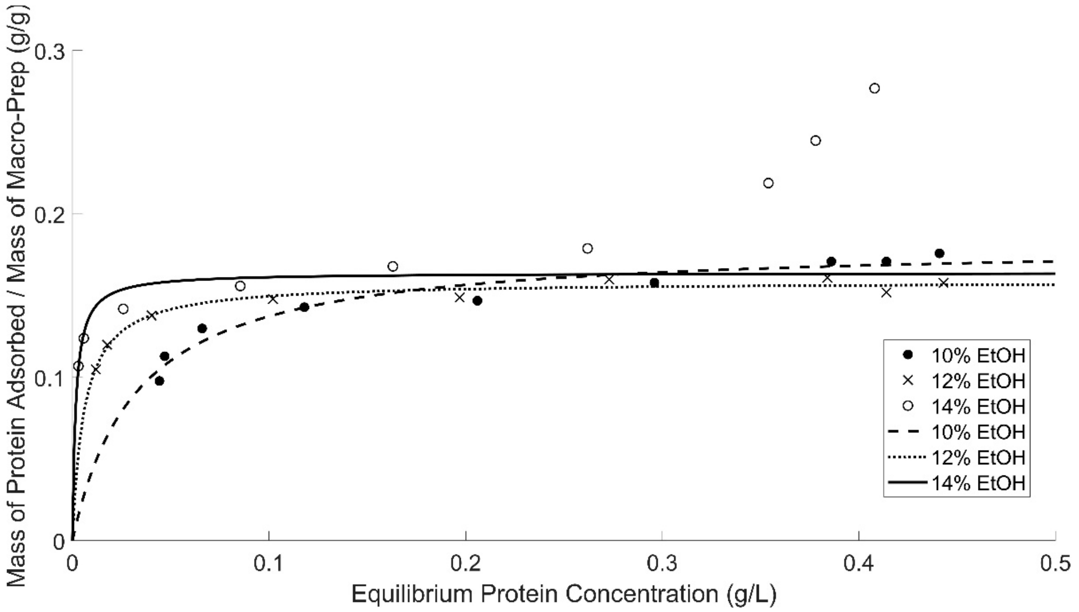

Figure S7. Macro-Prep's protein adsorption ability in model wine solutions with different ethanol concentrations characterized by UV-Vis spectrometer at 280 nm. Data for 14% *v*/*v* ethanol fitted only up to 0.17 (g BSA)/L.

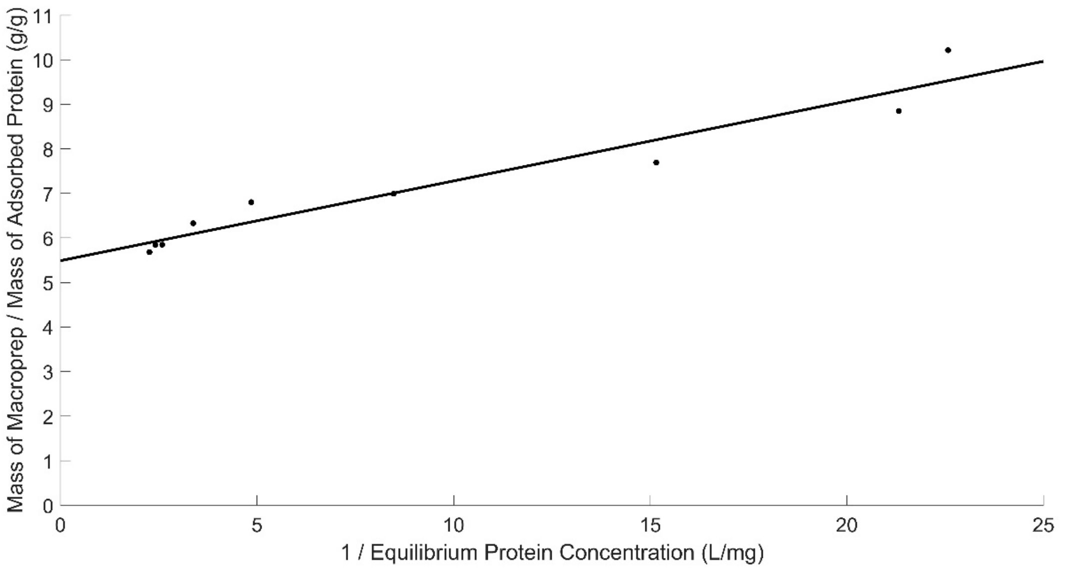

Figure S8. Lineweaver-Burk plot of Macro-Prep adsorption capacity in model wine solutions with an ethanol concentration of 10%. Data collected by using a UV-Vis spectrometer at 280 nm.

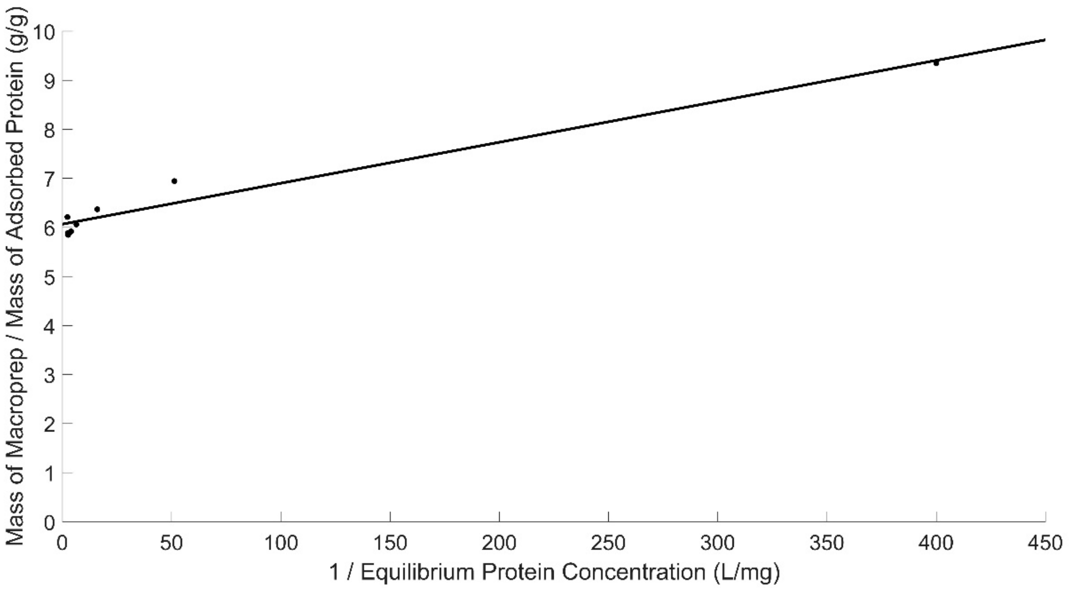

Figure S9. Lineweaver-Burk plot of Macro-Prep adsorption capacity in model wine solutions with an ethanol concentration of 10%. Data collected by using the Bradford protein assay and a UV-Vis spectrometer at 595 nm.

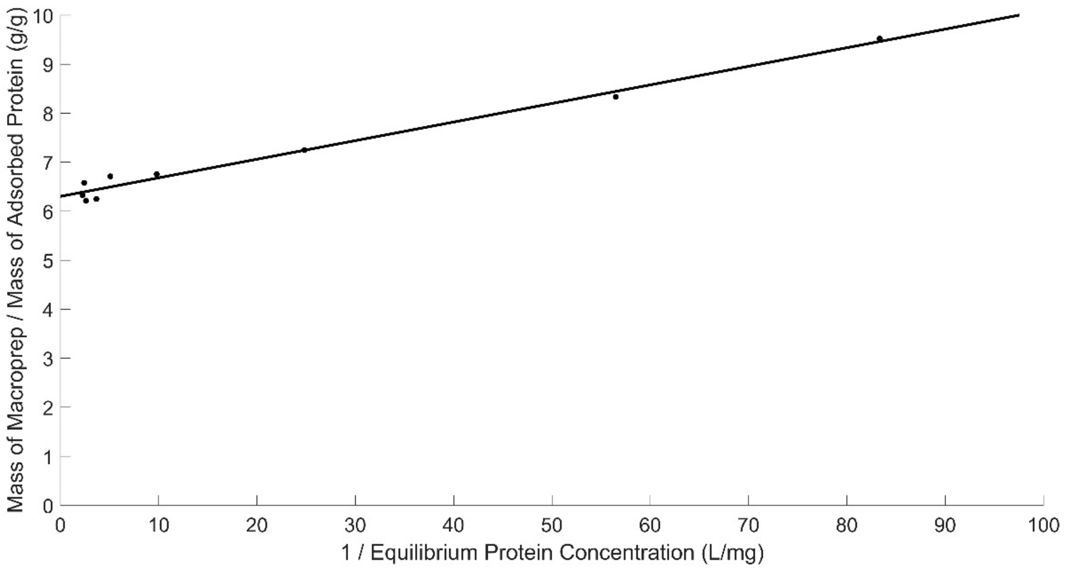

Figure S10. Lineweaver-Burk plot of Macro-Prep adsorption capacity in model wine solutions with an ethanol concentration of 12%. Data collected by using a UV-Vis spectrometer at 280 nm.

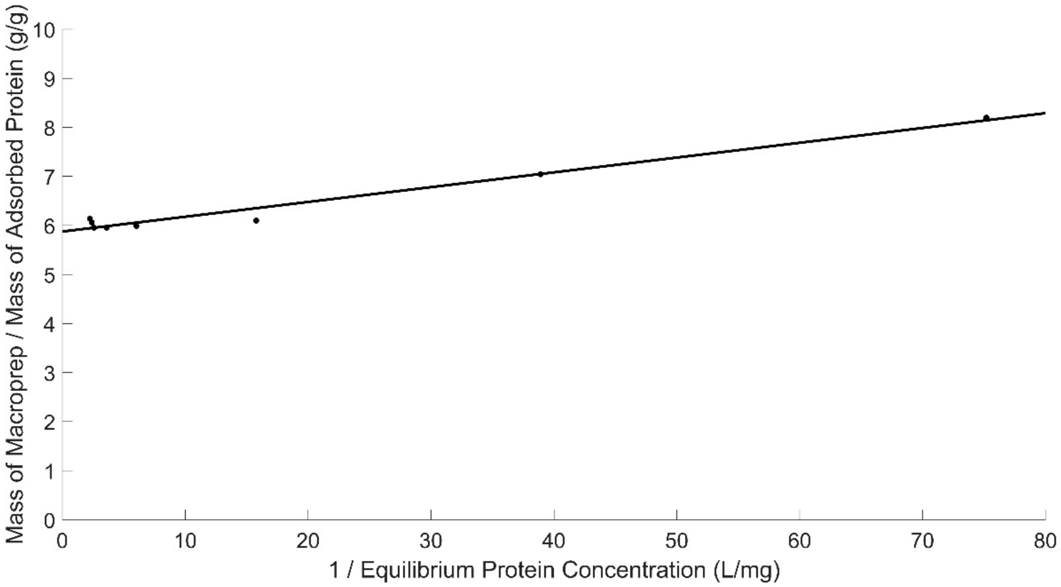

Figure S11. Lineweaver-Burk plot of Macro-Prep adsorption capacity in model wine solutions with an ethanol concentration of 12%. Data collected by using the Bradford protein assay and a UV-Vis spectrometer at 595 nm.

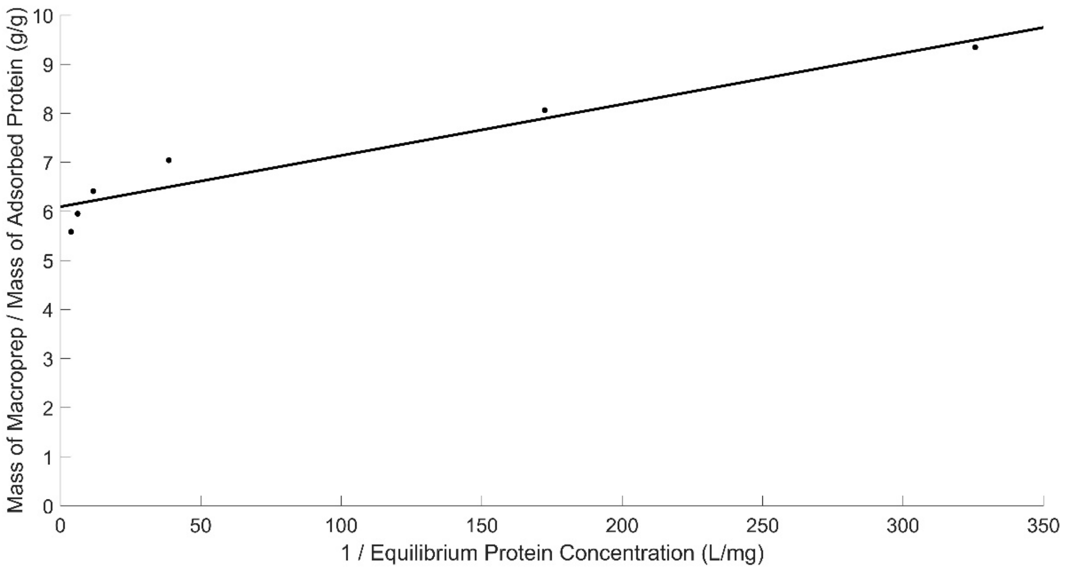

Figure S12. Lineweaver-Burk plot of Macro-Prep adsorption capacity in model wine solutions with an ethanol concentration of 14%. Data collected by using a UV-Vis spectrometer at 280 nm.

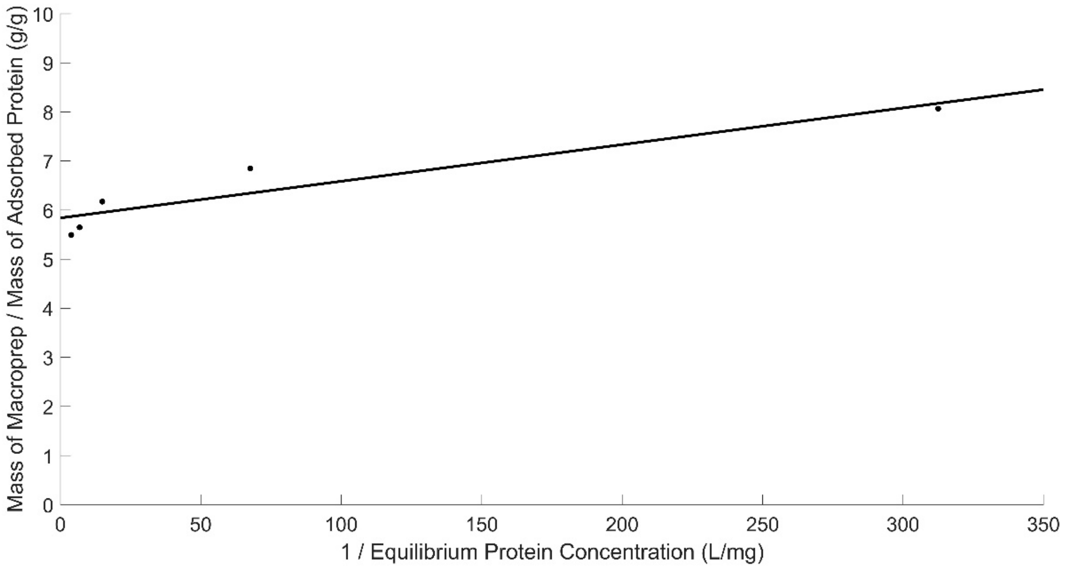

Figure S13. Lineweaver-Burk plot of Macro-Prep adsorption capacity in model wine solutions with an ethanol concentration of 14%. Data collected by using the Bradford protein assay and a UV-Vis spectrometer at 595 nm.

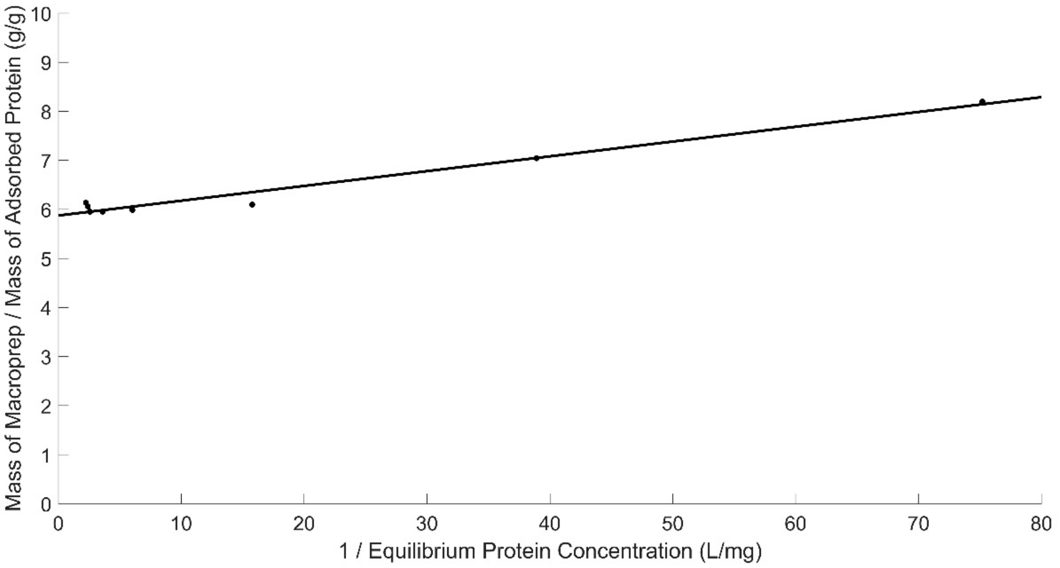

Figure S14. Lineweaver-Burk plot of Macro-Prep adsorption capacity in model wine solutions with no addition of caffeic acid. Data collected by using the Bradford protein assay and a UV-Vis spectrometer at 595 nm.

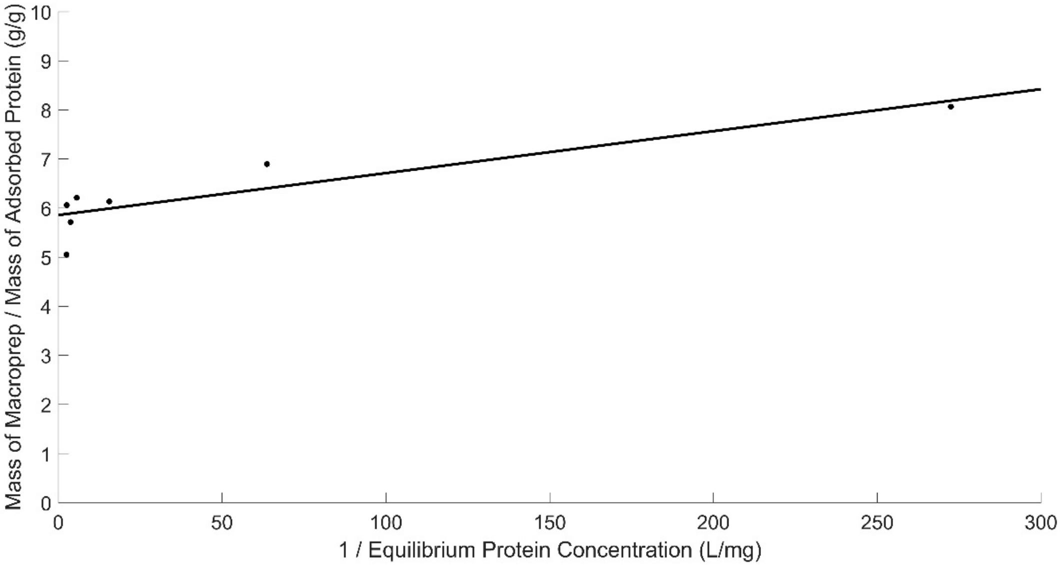

Figure S15. Lineweaver-Burk plot of Macro-Prep adsorption capacity in model wine solutions with addition of 75 mg/L caffeic acid. Data collected by using the Bradford protein assay and a UV-Vis spectrometer at 595 nm.

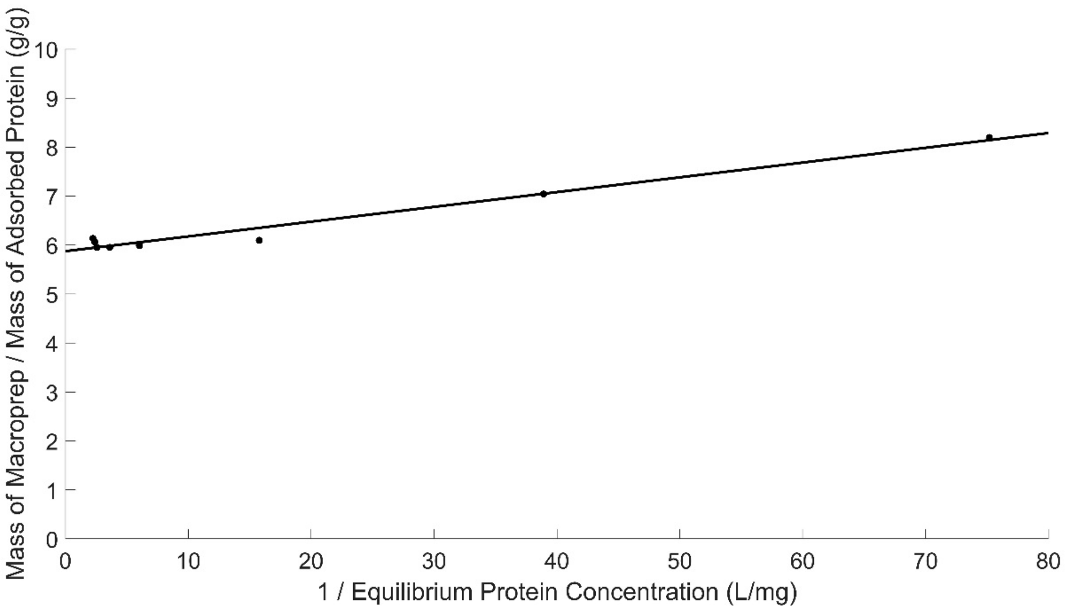

Figure S16. Lineweaver-Burk plot of Macro-Prep adsorption capacity in model wine solutions with no addition of catechin. Data collected by using the Bradford protein assay and a UV-Vis spectrometer at 595 nm.

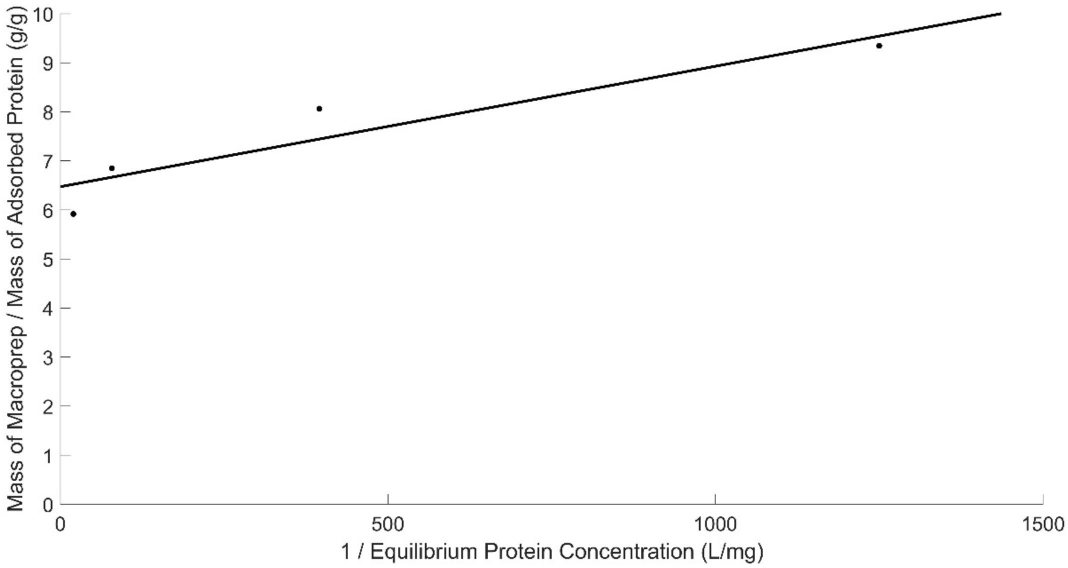

Figure S17. Lineweaver-Burk plot of Macro-Prep adsorption capacity in model wine solutions with the addition of 55 mg/L catechin. Data collected by using the Bradford protein assay and a UV-Vis spectrometer at 595 nm.

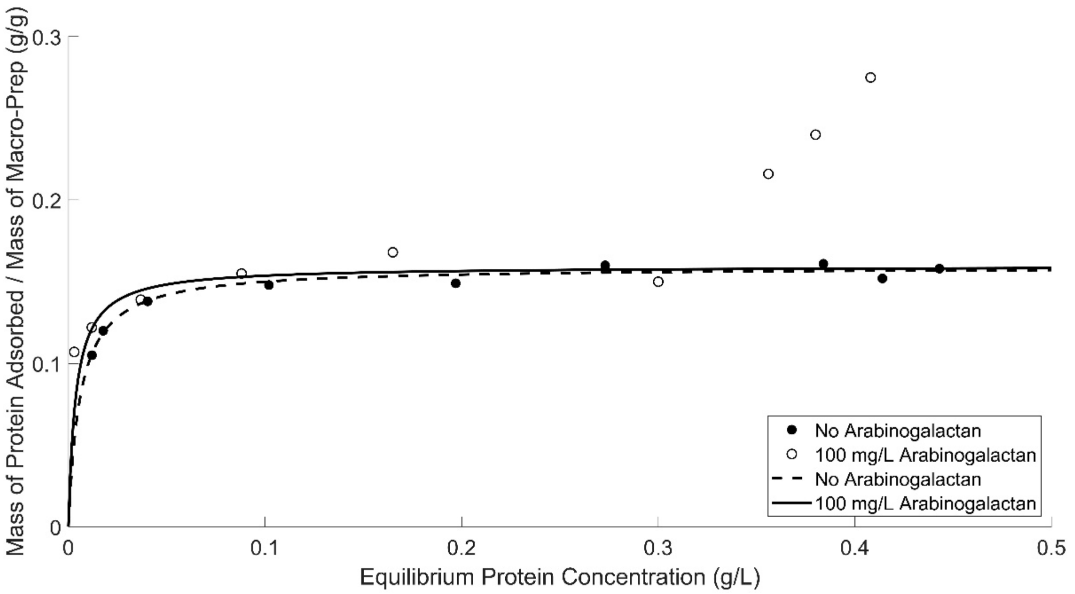

Figure S18. Macro-Prep's protein adsorption ability in model wine solutions with & without addition of arabinogalactan (AG) by using a UV-Vis spectrometer at 280 nm. The presence of the prototypical polysaccharide does not have a negative impact on the adsorption affinity or capacity of the ion-exchange resin for the model protein, BSA. Data for 100 mg/L of arabinogalactan is fitted only up to 0.3 (g BSA)/L.

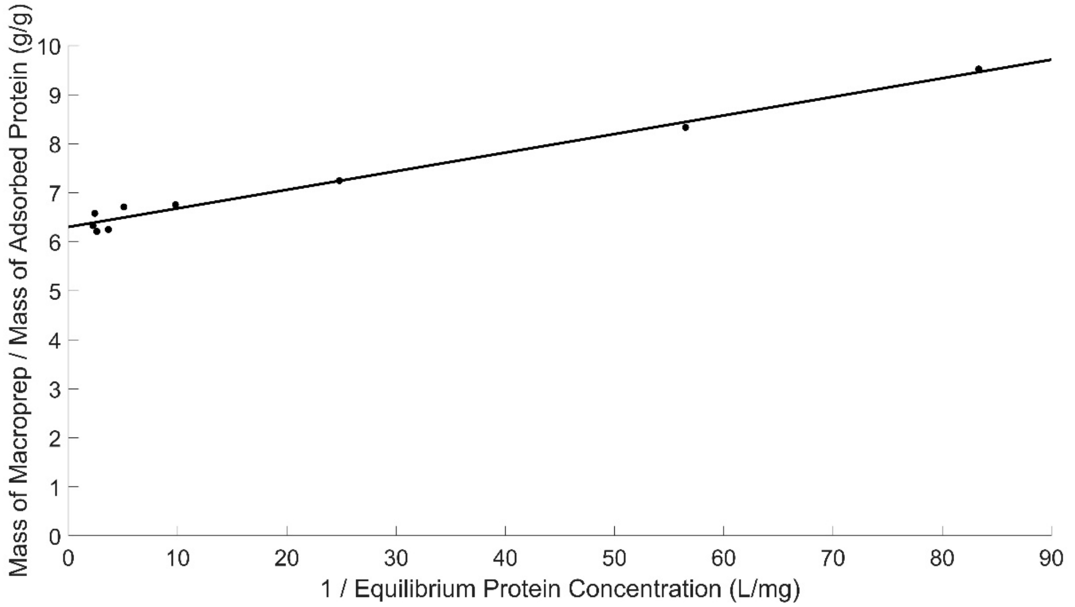

Figure S19. Lineweaver-Burk plot of Macro-Prep adsorption capacity in model wine solutions with no addition of arabinogalactan (AG). Data collected by using a UV-Vis spectrometer at 280 nm.

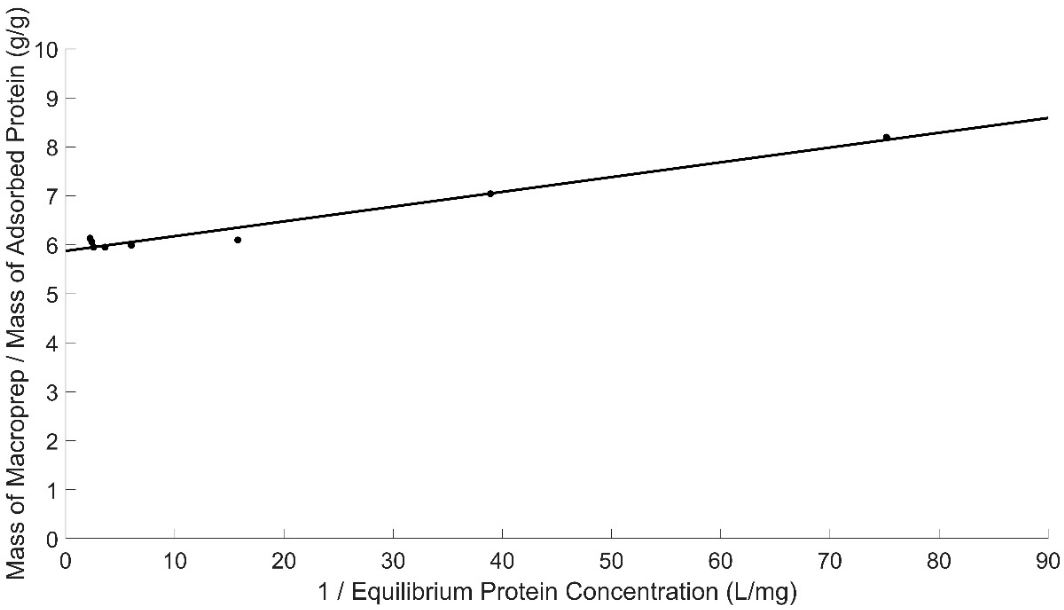

Figure S20. Lineweaver-Burk plot of Macro-Prep adsorption capacity in model wine solutions with no addition of arabinogalactan (AG). Data collected by using the Bradford protein assay and a UV-Vis spectrometer at 595 nm.

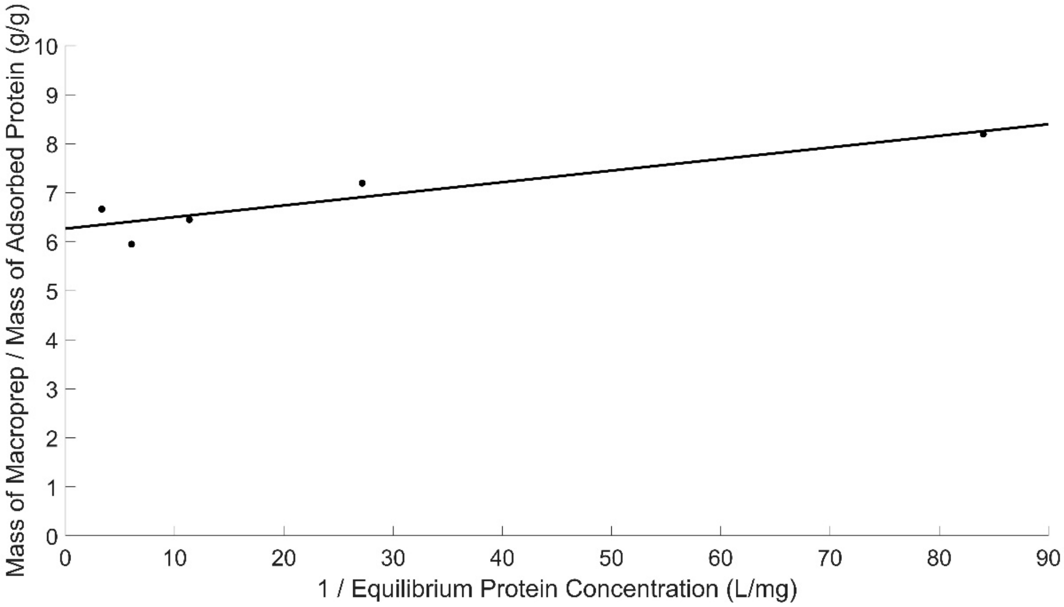

Figure S21. Lineweaver-Burk plot of Macro-Prep adsorption capacity in model wine solutions with the addition of 100 mg/L arabinogalactan (AG). Data collected by using a UV-Vis spectrometer at 280 nm.

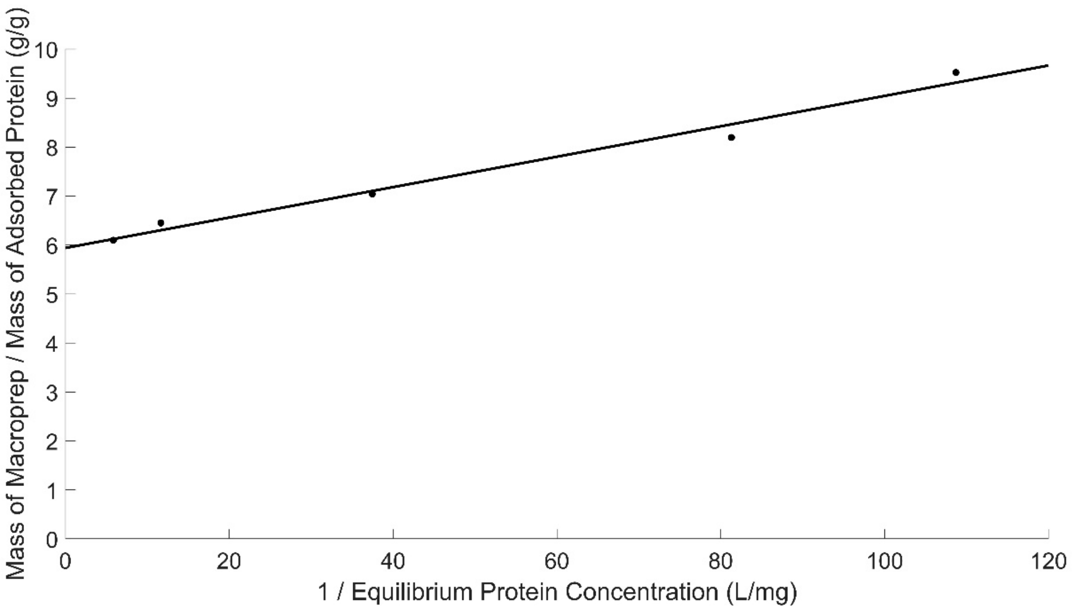

Figure S22. Lineweaver-Burk plot of Macro-Prep adsorption capacity in model wine solutions with the addition of 100 mg/L arabinogalactan (AG). Data collected by using the Bradford protein assay and a UV-Vis spectrometer at 595 nm.
